# Investigation of frequency and typing of human papillomavirus among genital warts using a reverse dot blot hybridization approach

**DOI:** 10.1186/s12879-022-07276-8

**Published:** 2022-03-22

**Authors:** Majid Zare-Bidaki, Mahmoud Zardast, Ali Nadjafi-Semnani, Mohammad Nadjafi-Semnani, Davod Javanmard, Shokouh Ghafari, Nahid Ghanbarzadeh

**Affiliations:** 1 Infectious Diseases Research Center, Birjand University of Medical Sciences, Birjand, Iran; 2 Department of Medical Microbiology, Faculty of Medicine, Birjand University of Medical Sciences, Birjand, Iran; 3 Deprtment of Pathology, Birjand University of Medical Sciences, Birjand, Iran; 4 Department of General Surgery, Zahedan University of Medical Sciences, Zahedan, Iran; 5 Department of Urology, Birjand University of Medical Sciences, Birjand, Iran; 6 Department of Gynecology and Obstetrics, Medical Faculty, Birjand University of Medical Sciences, Birjand, Iran

**Keywords:** HPV, Genotype, Cervical cancer, Wart, High risk, Low risk, Iran, South Khorasan

## Abstract

**Background:**

Human papillomavirus (HPV) is the most common sexually transmitted infection worldwide, affecting about 80% of women up to the age of 50. The persistent infection of high risk-HPV types (HR-HPV) is the leading cause of cervical cancer, the fourth most common cancer of women. Therefore, we aimed to evaluate the frequency and typing of HPV in the genital lesions in the Iranian population.

**Methods:**

This descriptive-analytic study was conducted on a population in the South-Khorasan province of Iran. All of the participants were sexually active and were checked for evident cervical warts. Biopsy samples were collected from various lesions, and all samples were tested for detection and genotyping of HPV using a reverse dot blot hybridization method (HPV direct flow CHIP).

**Results:**

In overall, 370 samples were evaluated; 10 cases (2.7%) were male and the rest were female. The mean age of patients was 33.3 ± 8.5 years, of which 48.1% were in the age range from 25 to 36 years. Among the samples, 345 (93.2%) were positive for HPV-DNA; the low risk HPV types (LR-HPV) and HR-HPV were identified among 80.9% and 15.5% of tissue samples, respectively. Among the LR-HPV, HPV-6, 11, 42 and 54 were the most common genotypes, and HPV-16 and 39 were prevalent HR-HPV types detected. The number of pregnancies, marriage age, and partner infection were not significantly related to the HPV types. Types 42 had a declining pattern toward aging, and HPV-11 was increasing toward aging.

**Conclusion:**

The number of samples with HR-HPV was rather high. Due to the greater frequency of infection in the age range of 25–35 years, it is advised that all individuals referred to gynecological clinics at gestational age be tested for HPV types.

## Introduction

Anogenital condyloma acuminata are genital lesions defined as wart caused by human papillomavirus (HPV) infections [1]. Genital warts include different types of flat or exophytic warts of the vagina and cervix in women, although, in men, condyloma acuminata could rise in the external genital such as the anogenital and penile area [2]. Usually, HPV infection is sub-clinical, and lesions spontaneously improve, resulting in mild disease. However, it may settle precancerous lesions that lead to an invasive form of cancer [3].

Cancers are the second leading cause of death in developed countries [4]. Hence, cervical cancer (CC) is the fourth leading cause of mortality among women globally and the first cause of death in the East, Middle, South, and West Africa [5]. Moreover, the vulvar and vaginal cancers account for about 4–7% of gynecologic cancers in women [6].

Oncogenic HPV-DNA is identified in many cancers such as the cervical, vulva, vaginal, vulvar intraepithelial neoplasia (VIN), and vaginal intraepithelial neoplasia (VAIN) [7–10]. Thus, it is nowadays a fact that the leading cause of these cancers worldwide is HPV [11]. This virus is a small double-stranded DNA virus belonging to the Papillomaviridae family [12]. HPV is commonly found in epithelial tissues and promotes proliferation of cells in the benefit of having E6 and E7 onco-proteins which have roles to disturb the tumor suppressors’ activity of p53 and pRB, respectively [13]. At present, more than 100 genotypes of HPVs are known [14], among which types 16 and 18 are the most common HPV types which were found in malignancies associated with this virus (notably Squamous Cell Carcinoma (SCC) [12, 15, 16]. Moreover, there are other HPV types which are high risk for CC including HPV-types 26, 31, 33, 35, 39, 45, 51, 52, 53, 56, 58, 59, 66, 68, and 82 [17].

Today, the preventive measures of primary (vaccination against HPV) and secondary (screening and typing of HPV along with treatment of precancerous lesions) have main roles to control the carcinogenic effect of the virus [5].

However, unlike developed countries, developing countries still lack an efficient and regular screening program, which is responsible for the rising prevalence of CC in these countries during the last three decades [18–22]. Though, it is evident that prevention, early detection, and timely treatment have an obvious effect on reducing CC-related death rather than any other cancers [23].

Screening tests are one of the preventive measures for cervical cancer. Pap smear screening is employed for nearly half a century to identify the precancerous lesions in advanced countries [24]. There are various techniques to detect HPV-DNA in clinical samples. However, polymerase chain reaction (PCR), in situ hybridization, and Southern blotting techniques are routinely employed [14].

Reports on HPV infection and typing in genital warts are limited. Most previous studies declare HPV types 6 and 11 as leading causes [25]; however, there are huge numbers of inconsistent results and evidences of roles of HR-HPV types in warts. Given the scarcity of data on HPV prevalence and genotypes in diverse parts of Iran, including the South Khorasan Province, this research looked at the frequency and variety of HPV types among various genital warts.

## Methods

### Patients and samples

This was a cross-sectional study conducted from October 2018 to March 2021 in the East of Iran, South Khorasan province. The population of the study were women and men referred to different gynecology clinics in the city of Birjand. A gynecologist selected patients with visible genital lesions, including wart types; one sample was taken from each patient. Condyloma acuminata defines as genital warts which are benign cauliflower-shaped lesions. Biopsy samples were given from the vulva, vagina, cervix, and external warts. The specimens were taken by a gynecologist and archived at the laboratory after investigating by an expert pathologist. The Ethics Committee approved the provisions of the research of Birjand University of Medical Sciences (Ethics code: IR.BUMS. 1398,168).

### Preparing samples

The tissue samples were fixed in formalin and passed via a tissue processor (Leica TP1020 Germany) and then embedded in paraffin. Afterwards, formalin-fixed paraffin embedded (FFPE) samples were subjected to sectioning by microtome (Leica RM2255 Germany). At first, 4 micron (4 µM) sections were undergone for Hemathoxilin and Eosin staining. Moreover, 10 other 4 µM sections were put into microfuge tubes for molecular assays. A separate blade was used for each sample, and necessary conditions and considerations were regarded to prevent carry over contamination of samples and/or sections.

### Detection and typing of HPV

According to the kit’s instruction, detection and typing of HPV were performed to the benefit of using the HPV Direct Flow CHIP test (Master Diagnóstica, Granada, Spain). This kit works in a reverse dot blot hybridization setting for multiplex detection and genotyping of HPV. In brief, the workflow of the above-mentioned kit is as follows:

First, tissue sections were subjected to removal of paraffin using an ethanol/xylene approach. Then, DNA was extracted using a tissue genomic DNA isolation kit (DNeasy Blood & Tissue Kit, Qiagen). Then, the extracted DNA was mixed with the Multiplex master mix of the HPV PCR reaction which was given in the content of the kit (HPV Direct Flow CHIP test, Master Diagnóstica, Granada, Spain). PCR Program was started with 5 min at 98 °C, followed by 5 cycles of 98 °C–42 °C–72 °C, and then followed by another 45 cycles of 98 °C–60 °C–72 °C.

**Fig. 1:**
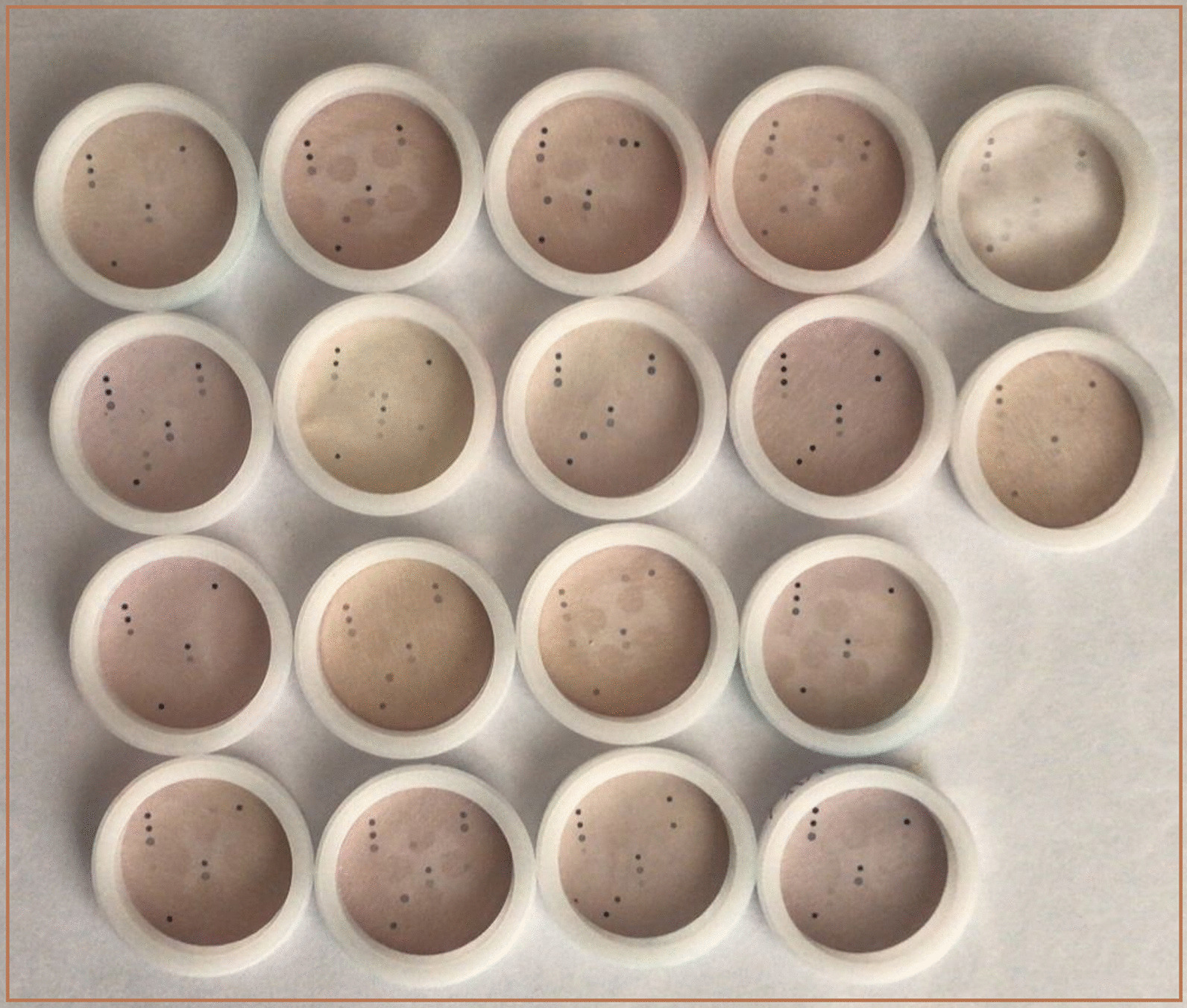
Positions of HPV DNA dots on microchip revealing HPV types in the genital samples of current study

The hybridization stage was performed by a full automated e-BRID System^®^ (Master Diagnostic Co, Spain). The microchip used in this method was blotted in 81 positions: 72 dot which is complementary with one single-strand DNA of each various 36 types of HPV, 5 dots for Blank or QC of chromogen, 2 for evaluation and control of correct extraction of DNA, and finally 2 dots for universal genotypes of HPV (Fig. 1).

### Statistical methods

The statistical analyses was performed using the Statistical Package for Social Sciences software version 25 (SPSS Inc, Chicago, IL, USA). Descriptive analysis was used to determine frequencies and percentiles. Chi-square test and Fisher’s exact test were used to test association and to compare between genotypes and age. The statistical significance was set at P < 0.05.

## Results

### Baseline information of population

**Fig. 2:**
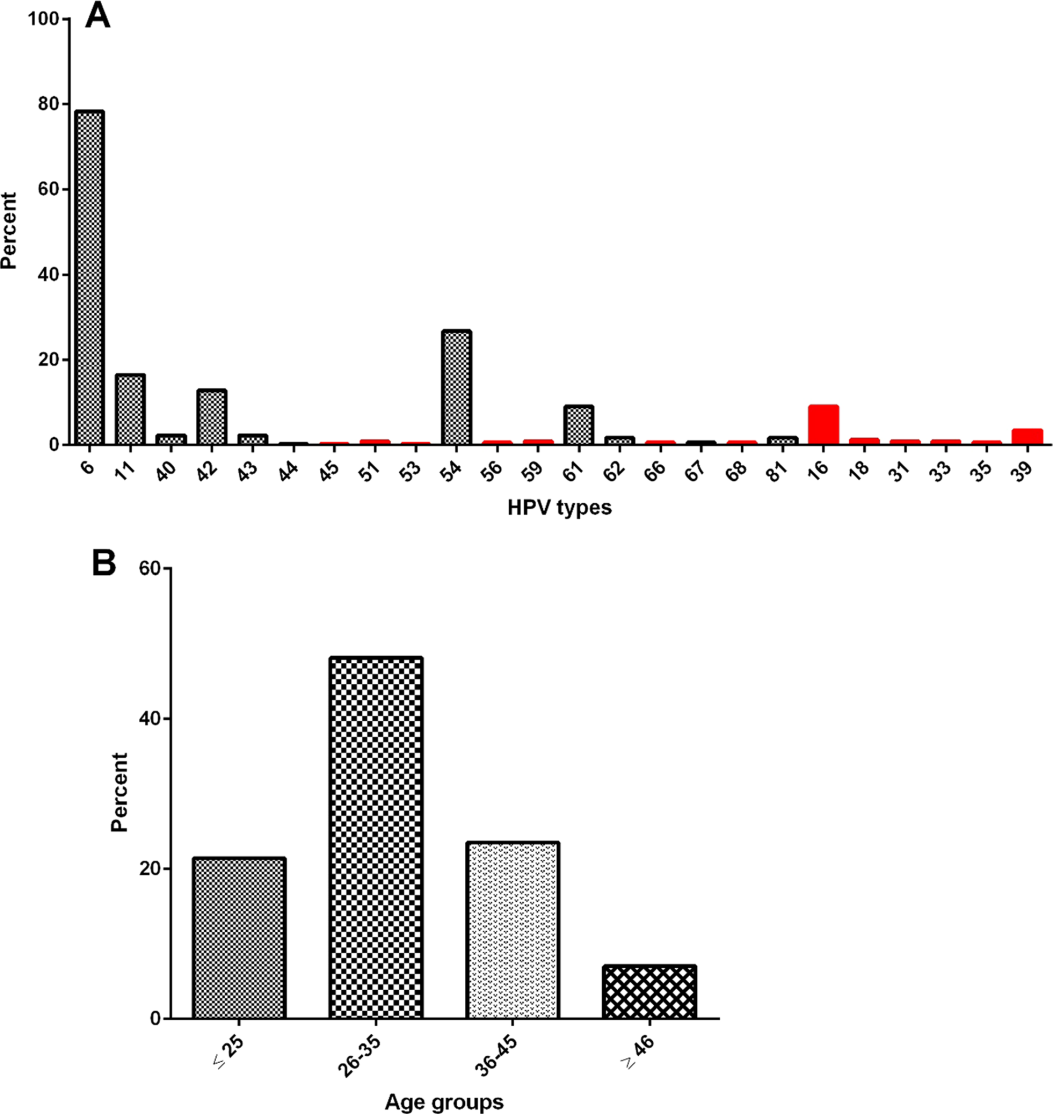
Distribution of different HPV types among the wart samples: LR-HPV types are labeled with black dashes, and HR-HPV have red bars (**A**); percentile of participants in the term of age categories was presented in **B**

In total, 370 samples of genital warts were collected from 370 patients. Among which, 10 cases (2.7%) were male, and the rest were female. The mean age was 33.3 ± 8.5 years old, with no significant difference among the genders which ranged from 14 to 88 years old (Fig. 2). Most of the participants were college-educated, and housewives (67.68% and 48.48%, respectively). The mean age of marriage was 22.46 ± 4.15 years, and 49.5% were married from 22 to 29 years. According to the baseline demographic information, 76% of patients had a history of 1 to 3 pregnancies.

### Prevalence and types of HPV

The result of the Direct Flow CHIP revealed 345 samples (93.2%) were positive for HPV-DNA, and the rest (6.7%) were defined as undetected HPV-DNA. The result of HPV genotyping showed that among the samples tested, type 6 (78.3%), type 54 (26.7%), type 11 (16.5%), and type 42 (12.8%) were the most prevalent LR-HPV types (Fig. 2). In total, HR-HPV types were identified among 53 samples (15.5%), of which type 16 (9%) and type 39 (3.5%) were the most prevalent. Single infections of HPV types 16 and 39 were detected among 5 and 11 samples, respectively. There was no statistically significant difference among the frequency of genotypes with the occupation, education level, number of pregnancies, age of marriage, and spouse infection of the participants (P > 0.05).

### Prevalence of mix HPV infections

In general, 158 samples (45.8%) were infected with only one HPV type (mono-type HPV infection), although 53.7% had more than one type simultaneously (mix-type HPV infections); among which 47% had a mix of two types, 6.1% three types and 0.6% had 4 types coincidently. HPV type 6 was most prevalent HPV type as mono-type HPV infection (68.4%), then followed by HPV types 11 (13.3%), 16 (4.4%), 54 (7%), 42 (1.9%) and 31 (1.2%). There were two samples with mix of 4 HPV types (Mix of types 6–11–16–35 and 6–43–53–54). Among the triple mix infections, the mix of types 6–11–42, 6–42–54, and 6–11–54 were observed in 4, 3, and 2 samples, respectively. Although there were more other triple infections that observed just in one tissue sample. Among the double HPV infections, mix of HPV types 6–42, 6–11, 6–16, 6–40, 6–54 were most prevalent with percentage of 8.1%, 4.9, 3.8, 1.4 and 16.8%, respectively (Table 1).

**Table 1:** Frequency and age related distribution of different pattern of mix infections with different HPV types

	N (%)	Mean age	< 25 N (100)	26–35 N (100)	36–45 N (100)	45 > N (100)	P^b^
Single type	158 (45.9)	32.1 ± 7.6	32 (20.3)	84 (53.2)	32 (20.3)	10 (6.3)	0.152
Coinfection	185 (54.1)	32.3 ± 8.3	42 (22.7)	81 (43.8)	49 (26.5)	13 (7)	0.152
Mix of two type	162 (47)	32.4 ± 8.3	36 (22.2)	71 (43.8)	42 (25.9)	13 (8)	0.068
Mix of 3 type	21 (6.1)	31 ± 7.8	5 (23.8)	10 (47.6)	6 (28.6)	–	0.088
Mix of 4 type	2 (0.6)	32.5 ± 7.6	1 (50)	–	1 (50)	–	0.130
HPV types in mix infections^a^							
6–42–54	3 (1.6)	28 ± 6.5	1 (33.3)	2 (66.7)	–	–	0.181
6–11–42	4 (2.2)	32.5 ± 8.8	1 (25)	2 (50)	1 (25)	–	0.150
6–11–54	2 (1.1)	29.5 ± 3.5	–	2 (100)	–	–	0.176
6–16–54	2 (1.1)	27 ± 7.1	1 (50)	1 (50)	–	–	0.141
6–11–39	2 (1.1)	40 ± 6.4	–	–	2 (100)	–	0.854
11–43	2 (1.1)	26.5 ± 3.5	1 (50)	1 (50)	–	–	0.541
6–42	28 (15.1)	30.9 ± 7.3	9 (32.1)	11 (39.3)	7 (25)	1 (3.6)	0.055
6–11	17 (9.2)	33.1 ± 9.1	2 (11.8)	10 (58.8)	3 (17.6)	2 (11.8)	0.091
6–18	2 (1.6)	23.5 ± 5	1 (50)	1 (50)	–	–	0.354
6–16	13 (7)	33.9 ± 7.5	2 (15.4)	4 (30.8)	6 (46.2)	1 (7.7)	0.354
6–40	5 (2.7)	39 ± 8.1	–	2 (40)	2 (40)	1 (20)	0.094
6–54	58 (31.3)	33.2 ± 7.9	9 (15.5)	28 (48.3)	18 (31)	3 (5.2)	0.078
6–43	3 (1.6)	32.3 ± 4.1	–	2 (66.7)	1 (33.3)	0	0.874
6–39	2 (1.1)	36.5 ± 16.2	1 (50)	–	–	1 (50)	0.954
6–51	3 (1.6)	35 ± 10	1 (33.3)	1 (33.3)	1 (33.3)	–	0.354
16–54	3 (1.6)	30.7 ± 10.6	1 (33.3)	1 (33.3)	1 (33.3)	–	0.358
6–66	2 (1.1)	35 ± 8.5	–	1 (50)	1 (50)	–	0.452
6–81	4 (2.2)	28.7 ± 3.9	1 (25)	3 (75)	–	–	0.864
11–54	4 (2.2)	33 ± 14.5	2 (50)	–	1 (25)	1 (25)	0.548
11–42	2 (1.1)	29 ± 9.9	1 (50)	–	1 (50)	–	0.879
39–54	2 (1.1)	28.5 ± 0.7	–	2 (100)	–	–	0.839

^a^The percent for mix infection are presented per total samples with coinfection

^b^The frequency of mix infection with HPV types 6–39–42, 6–39–54, 6–18–40, 11–16–40, 6–31–45, 6–16–56, 16–67–54, 6–62, 11–39, 6–68, 16–40, 42–59, 6–56, 42–54, 39–42, 43–54, 6–33 was one sample for each pattern

### HPV-types among different age groups

The mean age among participants with different HPV types was not statistically different (Table 2). Although, the pattern of some HPV infections was significantly different in regard to age groups. HPV types were considerably prevalent in the age group of 25–35 years, tough, the rate of HPV type 54 was growing in older ages and was more prevalent between 36 and 45 years. Moreover, HPV type 42 was more prevalent among ages lower than 25 years (P = 0.03). HPV type 6 was ubiquitously distributed in different age ranges, though, types 16 and 42 had a declining pattern toward aging, and HPV 11 was elevated in higher ages (Table 2).

**Table 2:** Frequency and age related distribution of different HPV types are presented

	HPV type	Number (%)	Mean age ± sd	< 25 N (%)	26–35 N (%)	36–45 N (%)	45 > N (%)	P^a^
Low risk HPV types	6	270 (78.3)	32.3 ± 7.8	55 (20.4)	129 (47.8)	70 (25.9)	16 (5.9)	0.152
	11	57 (16.5)	32.3 ± 8.2	12 (21.1)	28 (49.1)	12 (21.1)	5 (8.8)	0.068
	40	8 (2.5)	34.5 ± 9.9	1 (12.5)	3 (37.5)	3 (37.5)	1 (12.5)	0.314
	42	44 (12.3)	31 ± 8.1	15 (34.2)	17 (38.6)	9 (20.5)	3 (6.8)	0.030
	43	8 (2.3)	32.1 ± 10.6	2 (25)	3 (37.5)	2 (25)	1 (12.5)	0.128
	44	1 (0.3)	28	1 (100)	–	–	–	0.824
	54	92 (26.9)	32.5 ± 8.2	18 (19.6)	44 (47.8)	24 (26.1)	6 (6.5)	0.050
	62	6 (1.6)	30.5 ± 8.8	2 (33.3)	3 (50)	–	1(16.7)	0.258
	67	2 (0.6)	33.5 ± 9.2	0	1 (50)	1 (50)	–	0.523
	81	6 (1.6)	30.4 ± 7.6	3 (50)	3 (50)	–	–	0.442
High risk HPV types	16	31 (8.8)	31.6 ± 7.4	8 (25.8)	12 (38.7)	10 (32.3)	1 (3.2)	0.088
	18	4 (0.9)	24.7 ± 3.3	2 (50)	2 (50)	–	–	0.730
	31	3 (0.7)	27.3 ± 8.9	1 (33.3)	2 (66.7)	–	–	0.181
	33	1 (0.2)	22	1 (100)	–	–	–	0.150
	35	2 (0.5)	29.5 ± 6.3	1 (50)	1 (50)	–	–	0.176
	39	12 (3.7)	34.2 ± 10	2 (16.7)	5 (41.7)	3 (25)	2 (16.7)	0.091
	45	1 (0.3)	17	1 (100)	–	–	–	0.924
	51	3 (0.9)	35 ± 10	1 (33.3)	1 (33.3)	1 (33.3)	–	0.587
	53	1 (0.3)	40	–	–	1 (100)	–	0.875
	56	2 (0.6)	37.5 ± 5	–	1 (50)	1 (50)	–	0.658
	59	3 (0.9)	29.3 ± 6.4	1 (33.3)	2 (66.7)	–	–	0.458
	66	2 (0.6)	35 ± 8.5	–	1 (50)	1 (50)	–	0.635
	68	2 (0.6)	30 ± 4.2	–	2 (100)	–	–	0.847

^a^The p-value is for difference of prevalence among age groups

**Fig. 3:**
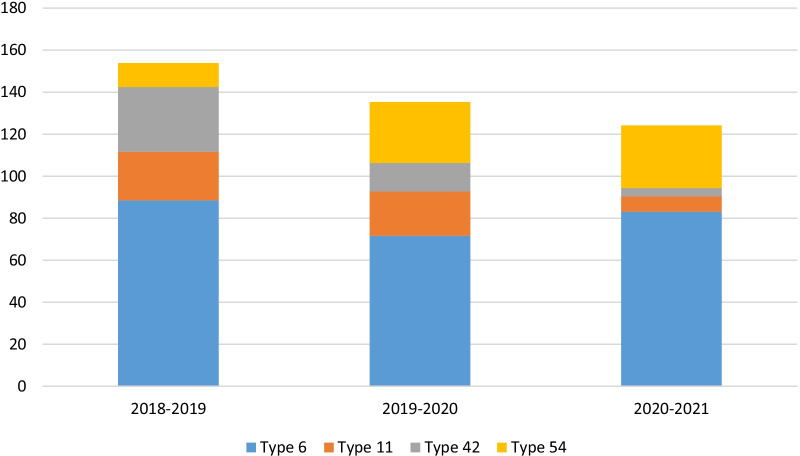
The incidence of different HPV type in 3 continuous year was presented as percent of each type

As sampling was followed per 3 continuous years, we compared the rate of different types among 3 years. It seems that the HPV type 6 is continually prevalent, type 11 is significantly declined and shifted to other low-risk types such as type 54 (P < 0.05) (Fig. 3).

## Discussion

It is widely accepted that HPV-DNA testing and typing are useful for screening CC. Previous studies indicated the higher sensitivity of HPV testing rather than cytology experiments due to the evaluation of the high-grade cervical intraepithelial lesions and invasive cancer [26]. Therefore, the superiority of the HPV-DNA testing and cooperation with the Pap smear test should be more concerned and be used in clinical situations.

In this study, a reverse dot blot hybridization assay, named as HPV Direct Flow CHIP was used to detect and typing of HPV. The overall detection rate of HPV-DNA among the wart samples was 93.2%, so, 6.7% defined as undetected HPV. Most of the previous similar works have been reported in this range [27–29]. No detection of HPV from wart samples is partly due to sample type, pre-processing, and formalin fixation and/or methodology used for detection of HPV.

According to our findings, HPV types 6, 11, 42, and 54 were the most prevalent LR-HPV types; however, HPV types 16 and 39 were prevalent HR-HPV types detected. There are limited studies around Iran that worked on genital warts; almost most of them reported HPV-6 as the leading cause of genital warts [30, 31]. Although there were studies that report the dominance of type 11 [29, 32], the sample size and methodology affect these figures. In south Khorasan province, Mousavi et al. recently reported 40.7% of HPV type 6 among wart samples that it declined with aging; however, in the current study, type 6 was consistent in all ages with 78.3% overall prevalence [33]. Moreover, the pattern of some types was changing over 3 years; types 11 and 42 declined and shifted to increasing of type 54. This finding is reasonable with minor differences from the result of Mousavi et al. [31].

In total, in this project, HR-HPV types were found in 15.5% of samples (including 9% single infection with HR and 6.5% coinfection with an HR), which is a considerable rate in terms of follow-up and cancer preventive programs. This rate is consistent with most of the previous reports on genital warts [28, 30]. Although, studies have reported HR-HPV as low as 1.5% [2], or high from 40 to 58.7% among genital warts [1, 34]. This sharp difference is mainly explained by the population type, geographic region, and the prevalence of associated risk factors. Some differences could be related to the technical issues of HPV detection and typing; most of the above-mentioned studies have used conventional PCR methods and/or sequencing. According to the extent of nucleotide heterogenecity among HPV types, the amplification and detection rate of all common HPV types might be reduced using limited numbers of primer pairs.

Another significant finding was the high prevalence of HPV types 42 and 54 in the current study. This figure was rarely reported from Iranian studies and seemed to be recently introduced and circulated in the Iranian population as LR-HPV [28, 33, 35, 36]. Although it was proven that the HPV types 6 and 11 are the most prominent types among genital warts [37], nevertheless, HPV types 42 and 54 were reported from urogenital regions [34]. Whilst HPV type 42 and 54 are proven as low risk HPV types [38], type 42 has been recently reported in association to the pathogenesis of Seborrheic keratosis-like lesion of genital tract [39].

The detection of more than one HPV type in the same sample, which is considered mix HPV infection and/or coinfection, was seen in 53.7% of HPV positive samples of this study (mix of two, three, and 4 HPV types was seen among 47%, 6.1% and 0.6% of HPV positive samples, respectively). This figure is in line with a just recently published study with a similar setting [2]. In reports with a similar setting, mix infections were varied from 13.4% [3], 33.8% [4], to 54% which were mostly used blotting and hybridization methods such as INNO-LiPA^®^ [5]. The high sensitivity of HC-2 and hybridization assays to detect HPV types were previously approved [6, 7]. The rate of coinfection has not been addressed in most of the previously reported studies [8, 9], and some reported it in very low levels [10–12], which were mostly used PCR-based assays.

The prevalence of genital HPV was significantly high in the age range from 25 to 35 years, which is in line with previous studies [40]. It was found that people over 25 years old have the highest rate of infection [41]. An investigation on 1000 women revealed the highest rate of HPV in cases aged 19–25 years [42]. However, Newall et al. found that the highest rate of HPV was in the age group of 30–39 years with the dominance of genotypes of 6, 16, and 18 [43]. As a result, the rising prevalence of HPV coincides with the onset of sexual activities, which may change somewhat between geographic locations. The above-mentioned statistic, as well as the relatively high occurrence of mono and coinfection with HR-HPV, demand that cancer-prevention programs pay greater attention. Moreover, HPV vaccine coverage in Iran seems reasonable, though, vaccination happens in later ages and is not completely adherent to the guidelines [13]. Besides, previous studies demonstrated that anal warts are often heterogeneously originated and could not be assumed to LSIL (Low-grade squamous intraepithelial lesion) [14]; hence, HPV typing is a useful tool to assign a precise classification and grading lesions.

## Conclusion

This study presents beneficial information on typing of HPV among genital warts that should be considered in the transmission rate of different HPV types, cancer prevention, and designing HPV types in vaccines. Regarding the 15.5% rate of HR-HPV among genital warts as a benign lesion, this figure is relatively high and needs more consideration. Hence, screening women in sexually active age is requisite for controlling HPV infection, as well as HPV typing.

## Data Availability

The supporting data for the finding of current study are available from the corresponding author upon a reasonable request.
